# The image-scratch paradigm: a new paradigm for evaluating infants' motivated gaze control

**DOI:** 10.1038/srep05498

**Published:** 2014-06-30

**Authors:** Michiko Miyazaki, Hideyuki Takahashi, Matthias Rolf, Hiroyuki Okada, Takashi Omori

**Affiliations:** 1 Brain Science Institute, Tamagawa University; 2 School of Social Information Studies, Otsuma Women's University; 3 Graduate School of Engineering, Osaka University; 4 These authors contributed equally to this work.

## Abstract

Human infants show spontaneous behaviours such as general movement, goal-directed behaviour, and self-motivated behaviour from a very early age. However, it is unclear how these behaviours are organised throughout development. A major hindrance to empirical investigation is that there is no common paradigm for all ages that can circumvent infants' underdeveloped verbal and motor abilities. Here, we propose a new paradigm, named the image-scratch task, using a gaze-contingent technique that is adaptable to various extents of motor ability. In this task, participants scratch off a black layer on a display to uncover pictures beneath it by using their gaze. We established quantitative criteria for spontaneous eye-movement based on adults' gaze-data and demonstrated that our task is useful for evaluating eye-movements motivated by outcome attractiveness in 8-month-olds. Finally, we discuss the potential of this paradigm for revealing the mechanisms and developmental transitions underlying infants' spontaneous and intentional behaviours.

Infants show various spontaneous behaviours from early in life, such as general movement^1^, self-exploration^2^, goal-directed behaviour^3^,^4^, motivated behaviour^5^,^6^, and intentional behaviour^7^,^8^. In this article, we refer to some behaviour as ‘spontaneous' when it is independent of external inputs but is driven by lower- or higher-level internal (e.g. non-reflective, habitual, motivated, or intentional) states. This is a broad term including various behaviours from non-meaningful bodily movement to intentional behaviour. In the early stages of life, infants move their hands and arms arbitrarily and independently of external stimuli, which is referred to as general movement^1^. Several months later, infants gradually begin to generate actions that are driven by their own motivation or intention^8^ (i.e. intentional control).

Clarifying when and how these spontaneous behaviours pass from lower to higher levels is one of the great issues of developmental science. In particular, it is important to illustrate using quantitative measurements how these behaviours develop. In the case of experiments with adults, verbal reports or complex hand manipulation are usually used to evaluate the inner state of the subject^9^,^10^.

However, infants' large variance of motor abilities throughout various developmental stages prevents us from using such measures for cross-age comparisons^11^. A non-verbal experimental method and criterion that is adaptable to various ages, producing comparable measurements across ages, is required for a precise understanding of the process of emergence of spontaneous behaviour.

Here, we propose a new experimental task using the gaze-contingent technique (i.e. online interactive eye tracking) to quantify spontaneous behaviour, including higher-order processing (e.g. motivation or intention), in infants. The task was named the image-scratch task (Fig. 1A). In this task, colourful, attractive pictures covered by a black layer are presented on a display equipped with an eye tracker, and participants are able to scratch off the black layer by gaze control and expose the picture beneath the layer. We hypothesised that, if the participants become aware of the novel contingency between their gaze coordinate and the change of stimulus, they will try to scratch off the black layer by controlling their gaze, being motivated by exploring the hidden, colourful picture.

The gaze-contingency paradigm has the advantage of being able to investigate the development of spontaneous behaviours in infants regardless of the variance of motor abilities in infancy because voluntary gaze control develops earlier than limb control^12^,^13^,^14^,^15^. This task is expected to adapt to various ages in the same manner and become a promising method for quantitative cross-age comparison of spontaneous behaviour in infants.

To demonstrate the usefulness of the image-scratch task for evaluating the extent of spontaneous, motivated behaviour, we examined whether 8-month-olds can control their gaze spontaneously to scratch the layer from the screen, by comparing the infant data to adults' experimental results on the same task. We established quantitative measures for spontaneous gaze control based on the adults' experimental results. We then evaluated the eye movements of the 8-month-old infants by these measures (Experiment 1).

Several previous studies have used the gaze-contingent technique^16^,^17^. However, none of these studies has managed to distinguish infants spontaneous gaze control from reflex-based eye movements induced by the mere visual saliency of the stimuli. To evaluate whether the eye movements of infants are truly driven by outcome attractiveness regardless of the visual saliency factor, we conducted two additional control experiments. Firstly, we reduced the outcome attractiveness in Experiment 2 by using monochromatic (greyscale) images as hidden pictures instead of colourful pictures (See Supplemental Information) in order to investigate whether the infants were truly motivated to scratch off the black layer and to expose the hidden pictures. Secondly, to examine whether infants were truly influenced by the gaze-scratch contingency instead of mere visual saliency, we performed a yoked control experiment using the presentation of a movie that showed exactly what had been on the screen of other infants classified as showing spontaneous scratching (See Supplemental Information, Experiment 3).

## Results

### Experiment 1: efficient measurement of spontaneous eye movement for scratching in adults and evaluation of eye movement in 8-month-old infants

To establish a measure of spontaneous gaze control in adults, we compared the eye movements of those adults who subsequently reported that they had discovered the action–outcome contingency and scratched the layer with intention (adults showing spontaneous scratching) with the behaviour of those who reported that they did not notice the rule and simply looked at the display without any intention (adults showing passive viewing). Subsequently, we evaluated the eye movement of infants using this measure.

**Figure 1:**
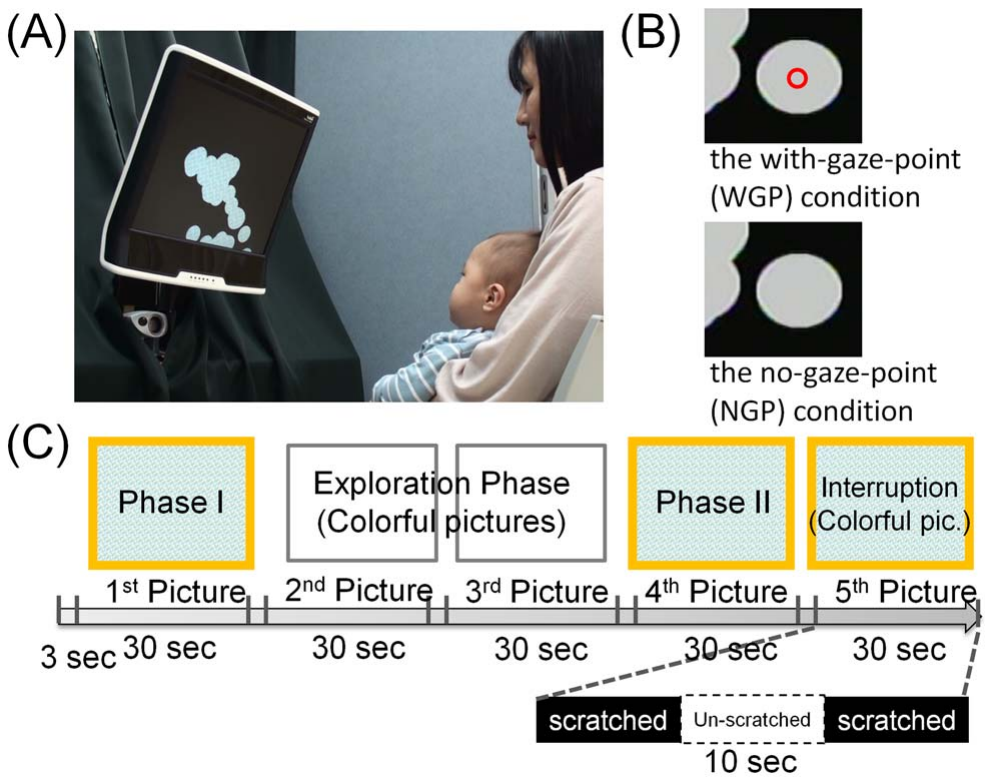
The image-scratch task. (A) Participants are able to scratch off the black layer using their gaze. This photo courtesy of Michiko Miyazaki with permission of the participants. (B) Two experimental conditions. In the with-gaze-point (WGP) condition, a small red circle is presented simultaneously at the point where the participants' gaze is. In the no-gaze-point (NGP) condition, the red circle is not presented. (C) The task includes five trials; each displaying one picture for 30 s. Pictures in trials 1 and 4 are solid simple geometric patterns (Phases I and II). To reveal subjective prediction errors indicating acquisition of the gaze-scratch contingency, a 10-s interruption phase, in which eye movements cannot scratch off the black layer, is inserted in the middle of the fifth trial.

To collect sufficient samples of adults in both spontaneous scratching and passive viewing, we prepared two conditions that differed in difficulty (easy/difficult-to-detect contingency). In the easy condition, a small red circle was presented simultaneously at the point where the participants' gaze was, making it easier for the participants to find the gaze–scratch contingency (the with-gaze-point [WGP] condition). In the difficult condition, the red circle was not presented (the no-gaze-point [NGP] condition) (Fig. 1B).

Forty adults participated in this study. The adults were instructed to sit in front of the display and look at it, with no further instructions regarding the task. In the image-scratch task, five pictures were presented one-by-one on the display for 30 s each (Fig. 1C). In the last trial, a 10-s interruption phase was inserted within 20 s of normal operation, the onset of the interruption being chosen randomly for each participant between 7 s and 10 s after beginning of the trial. In this phase, the eye movements of participants did not scratch off the black layer, even if a fixation was detected. Instead, the image remained unchanged. Before and after the interruption phase, gazes scratched off the image as in all other trials. After the task, the adults were asked to complete a questionnaire asking whether they noticed the gaze–scratch contingency and scratched off the black layer intentionally.

#### Questionnaire report of spontaneous eye movement in adults

We categorized those individuals who satisfied the following conditions as the spontaneous group: the individual who felt they were controlling their gaze intentionally, plus they detected the gaze-contingency. The number of those who detected the gaze-contingency was 27. Twenty-four of them were controlling the red circle intentionally.

The ratio of adults showing spontaneous scratching in the with-gaze-point condition (16 out of 19) was significantly higher than that observed in the no-gaze-point condition (8 out of 17; Fisher's exact test; *χ*^2^ = 5.57, *df* = 1, *p* = 0.033, φ = 0.23; Table 1).

#### Eye movements reflecting spontaneity in adults

**Figure 2:**
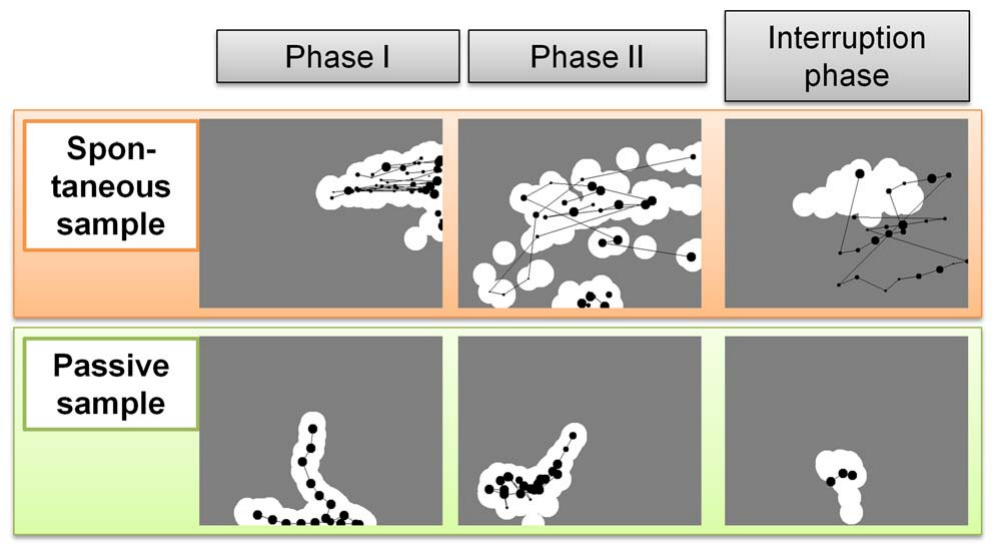
Typical eye-movement trajectories (A) adult showing spontaneous scratching and (B) adult showing passive viewing. The exposed areas are represented by the white circles. The exposed areas in Phase II are larger for the spontaneous adult than for the passive adult. The fixation duration is represented by the size of circles, with larger circles indicating longer fixation time. The fixation circles obtained in Phase II are smaller for the spontaneous adult than for the passive adult. In the interruption phase, the spontaneous adult gazes at the unscratched black layer and moves her/his gaze rapidly, whereas the passive adult only gazes at the exposed area.

The eye movements of adults showing spontaneous scratching contrasted with the eye movements of adults showing passive viewing (typical eye movements of both groups are shown in Supplemental Videos S1 and S2). Figure 2 shows typical eye movement trajectories of adults showing spontaneous and passive viewing. The left and middle panels of Figure 2 show that the exposed areas in the second phase (Phase II) are larger for the spontaneous adult than for the passive adult. The right side of Figure 2 depicts samples of the gaze trajectories of both an adult showing spontaneous scratching and an adult showing passive viewing during the interruption phase. The spontaneously scratching adult looked at the unscratched black layer and moved her/his gaze rapidly, whereas the adult showing passive viewing only gazed at the already exposed area. We interpret rapid, black-directed eye movement during the interruption phase as exploratory behaviour caused by a prediction error that indicates acquisition of the gaze-scratch contingency. We define the exploration rate during this phase as the proportion of gazes aimed at the black layer in relation to all gazes directed towards the display.

**Figure 3:**
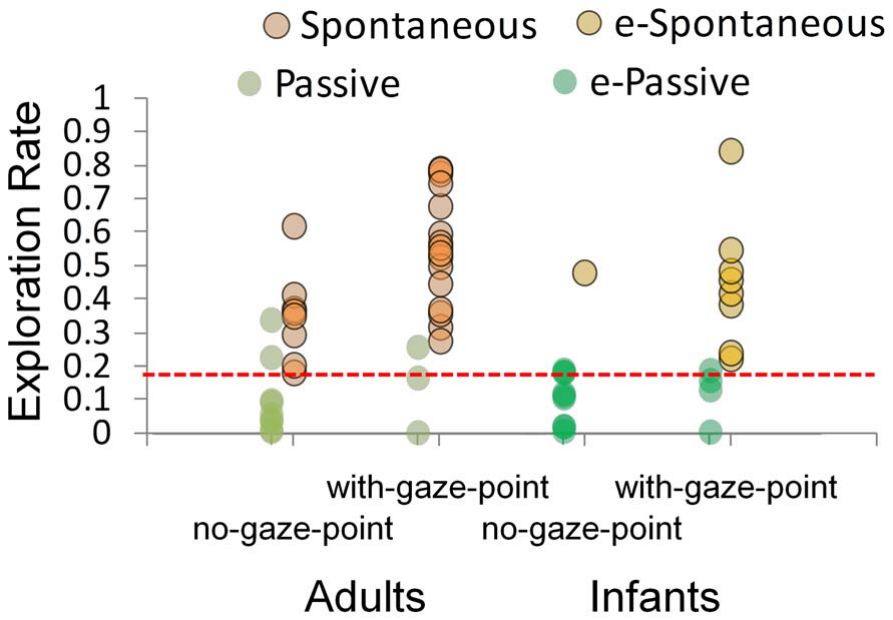
Exploration rate during scratch interruption. Individual plots of gazing at the black area during the scratch interruption phase for each participant group (adults/infants) and condition (no-gaze-point/with-gaze-point). The dark-green, dark-orange, light-green, and light-orange plots indicate adults in passive viewing, adults in spontaneous scratching, infants in passive viewing, and infants in spontaneous scratching, respectively. The dotted line refers to the threshold established for the discrimination of intentionality in adults, which is used for the estimation of spontaneity in infants.

Figure 3 shows individual plots of the exploration rate during the scratch interruption phase for each participant group (adults/infants) and each condition (no-gaze-point/with-gaze-point). In the two plots on the left side of Fig. 3, the dark-orange plots refer to the exploration rates of the spontaneously scratching adults. The dark-green plots refer to those of the passively viewing adults. We combined the data from the no-gaze-point and with-gaze-point conditions based on these latter two categories. Results show that the exploration rates of the adults showing spontaneous scratching are significantly higher than those of the adults showing passive viewing (*t*[33] = −7.54, *p* = 0.00000001, *r* = 0.80). To find a criterion that provides the best discrimination of adults showing spontaneous scratching from adults showing passive viewing, we tested how well the exploration rate could predict the questionnaire outcomes by means of a simple threshold decision. Therefore we tested a range of different thresholds and evaluated the rate of correct classification for each threshold value. In order to estimate the optimal threshold we interpolated the resulting curve by means of a third order polynomial and searched for its peak value within admissible thresholds. The optimal performance was reached at 88.9% correct classifications for a threshold value 0.187. Hence, defining a threshold of 0.187 on the exploration rate allows to distinguish adults showing spontaneous scratching from adults showing passive viewing with 88.9% accuracy. We verified our method ex post by means of a leave-one-out cross validation, which showed the same classification rate (88.9%) as the direct data-driven estimation, which shows the robustness of the estimation.

Cases of high exploration rates could also be explained by the scratched area being rather small at the onset of the interruption. To examine this issue, we tested whether the exploration rate was correlated to the size of the scratched area at the onset of interruption. We found no correlation between the scratched area and the exploration rate both in adults (*r* = −.11) and infants (*r* = −.02). These results suggest that high exploration rates were not caused by the larger black area at the onset of interruption.

The analysis of the adult eye movements yielded another significant index of spontaneous eye movement in addition to the exploration rate. This index is the difference in the size of the scratched area from the first trial (Phase I) to the fourth trial (Phase II), which is here termed the “intra-individual change of gaze control” from former to latter trials (see details regarding the estimation of eye movement in infants). As can be seen in the left and middle panels of Fig. 2, the exposed areas in Phase II are larger for adults showing spontaneous scratching than they are for adults showing passive viewing. Although the scratched area clearly differentiates between the adults showing spontaneous scratching and passive viewing, the index does not exhibit an optimal threshold that would allow a prediction of the questionnaire reports with high accuracy.

Taken together, the results obtained in the adult experiments show the existence of two indices for spontaneous eye movement: size of scratched area and exploration rate. Among them, the exploration rate in the interruption phase has the best threshold for a high-accuracy prediction of the questionnaire reports (88.9%). On the other hand, the size of the scratched area has no threshold allowing a better than 80%-accurate prediction of the questionnaire reports. Thus, we conclude that the exploration rate is the best predictive indicator of spontaneous eye movement.

#### Estimation of eye movement in infants

Twenty-two 8-month-old infants participated in either the with-gaze-point or the no-gaze-point condition. We adopted the threshold that was established in the adult experiments to evaluate the eye movements of the infants (see Fig. 3; above threshold, infants classified as showing spontaneous scratching; below threshold, infants classified as showing passive viewing). Typical eye movements of spontaneous- and passive-classified infants can be seen in the Supplemental Information (Supplemental Videos S3 and S4). The ratio of infants showing spontaneous scratching in the with-gaze-point condition (8 out of 12) is significantly higher than that in the no-gaze-point condition (1 out of 9; see Table 1; Fisher's exact test; *χ*^2^ = 7.25, *df* = 1, *p* = 0.011, φ = 0.58). This pattern is consistent with that observed in the examination of adults. This result suggests that most infants in the with-gaze-point condition show eye movement similar to the adults who executed the image-scratch task spontaneously. To validate our findings further, we analysed whether the size of the scratched area supports our categorisation of infants. As mentioned above, the typical adult showing spontaneous scratching scratched off a larger amount of the black layer by shifting her/his fixation quickly compared to the typical adult showing passive viewing (see Fig. 2). We found similar tendencies in the size of the scratched area between the spontaneous and passive participants in both the adult and infant groups.

**Figure 4:**
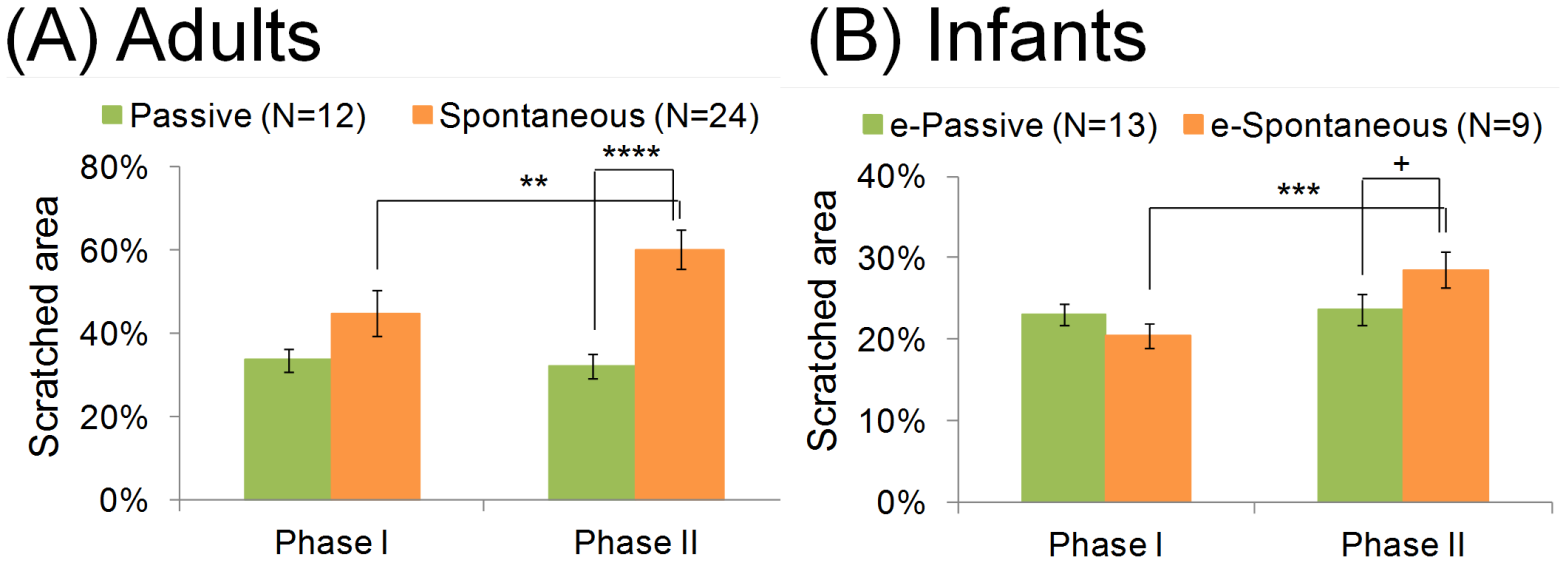
Mean proportion of the scratched area in Phases I and II. (A) In adults and (B) infants. The error bars indicate standard error.

A two-by-two, (Spontaneity: spontaneous vs. passive) × (Phase: Phase I vs. Phase II) ANOVA analysis was conducted on the size of the scratched area, revealing a significant interaction between Spontaneity and Phase in both adults and infants (adults, *F* [1, 34] = 4.77, *p* = 0.036, *η*^*2*^ = 0.14; infants, *F* [1, 20] = 6.29, *p* = 0.02, *η*^*2*^ = 0.32; see Fig. 4). From the simple main effect test, the effect of Spontaneity in Phase I is not significantly different between the two groups (adults, *F* [1, 34] = 2.12, *p* = 0.21, *η*^*2*^ = 0.06, non-significant [n.s.]; infants, *F* [1, 20] = 1.94, *p* = 0.24, *η*^*2*^ = 0.09, n.s.), whereas in adults, this effect is significantly different between the groups in Phase II. In infants, this effect is marginally significant (adults, *F* [1, 34] = 16.34, *p* = 0.001, *η*^*2*^ = 0.32; infants, *F* [1, 20] = 3.21, *p* = 0.18, *η*^*2*^ = 0.14). The effect of Phase in Passive group is not significantly different between the two groups (adults, *F* [1, 34] = 0.07, *p* = 0.80, *η*^*2*^ = 0.002, n.s.; infants, *F* [1, 20] = 0.07, *p* = 0.79, *η*^*2*^ = 0.003, n.s.), whereas this effect is significantly different between the groups in the Spontaneous group (adults, *F* [1, 34] = 8.02, *p* = 0.016, *η*^*2*^ = 0.19; infants, *F* [1, 20] = 14.52, *p* = 0.004, *η*^*2*^ = 0.42).

Furthermore, the proportion of short fixations shows similar tendencies in both adults and infants (see Supplemental Information, Figure S1). In the second phase, the proportion of short fixations is higher in the spontaneous group than in the passive group.

## Discussion

The present study proposes a new paradigm named the image-scratch task. It uses a gaze-contingent technique for quantifying spontaneous behaviours by young infants to adults by means of common measurements. Quantified behaviours include those driven by higher-order processes (i.e. motivation or intention). Based on results from adults, we have established a quantitative measure of spontaneous gaze control in infants and have demonstrated that at 8 months, an infant's gaze is driven, at least partially, by their motivation (see details in Supplemental Information, Experiment 2). We found that the exploration rate during the interruption phase is an efficient measure for detecting spontaneous gaze in adults. We optimised a threshold on this rate for predicting participants' questionnaire reports, with good results. Interestingly the ratio of spontaneous adults was significantly higher in the with-gaze-point condition than in the no-gaze-point condition.

We adopted this threshold to estimate whether an infant scratched the black layer off by motivation. The exploration rate of half of the infants was over the threshold in the with-gaze-point condition (but not in the no-gaze-point condition) and the validity of our method was further supported by the mean size of the scratched area in spontaneous infants, which was significantly higher than the size of the area scratched by passive infants (see Experiment 1). From these results, we can conclude that 8-month-old infants can control their gaze for exploring the picture behind the black layer.

Our method is based on a direct comparison of eye movements between infants and adults. However, a possible limitation of this direct comparison is that infants' eye movements could be only superficially similar to those of adults. That is, similar eye movements could be driven by mechanisms (e.g. reflective eye movement) different from those of adults, mechanisms that are not directly accessible since infants cannot report directly as adults can. To check for this issue, we conducted two additional experiments (see details in Supplemental Information, Experiments 2 and 3). In Experiment 2, we evaluated whether infants' eye movements were truly derived from the motivation for exploring attractive pictures. If infants' eye movements were not passive, but spontaneous, the size of the scratched area should be reduced when the attractive colourful pictures were replaced by monotone grayscale images (non-attractive condition). The results suggest that the size of the scratched area is indeed reduced in the non-attractive condition, whereas the exploration rate remains high. In Experiment 3, we examined the possibility that infants' spontaneous eye movements are driven by reflex-based gaze evoked by the saliency of visual images. Most infants categorised as spontaneous were continuously exposed to a red circle indicating their gaze point during the image-scratch task. The movement and visual saliency of the red circle possibly evoked reflex-based gaze and led to their different gaze pattern. To exclude this possibility, we performed a yoked control using playback movies of the screen contents seen by the spontaneous infants, but in a non-contingent condition. The result supports the contention that the spontaneous infants controlled their gaze independently of the saliency of the visual stimulus. Taken together, these findings suggest that 8-month-old infants spontaneously control their gaze, which is driven by motivation in our task.

A seminal study by Wang et al. using the gaze-contingent paradigm has addressed the quantification of infants' gaze control using a similar approach^16^. In their task, infants could press the button on an eye tracker's display by their gaze to play an attractive movie. The authors demonstrated that 6- to 8-month-old infants would choose a controllable button over an uncontrollable one, and that their eye movements are similar to the eye movements of adults who realise the contingency between their gaze and movie playing. Wang et al.'s approach is innovative; however, the similarity of eye movements between the infants and the adults who detect the contingency may be no more than a superficial approximation and may not reflect a common mechanism.

Our findings suggest that the 8-month-olds can control their gaze, which is driven by their motivation. However, our current findings have not decided the issue of whether infants actually have an explicit expectation of the outcome of their own behaviour^17^,^18^,^19^. Several previous studies have shown that younger infants can execute motivated behaviours such as goal-directed behaviours^20^,^21^. However, such behaviours are not necessarily to be interpreted as being accompanied by explicit expectations^22^. Instead, they can be explained as habitual responses^23^. For example, after learning that pulling a supporting blanket is needed for reaching an out-of-reach toy, 16- and 24-month-old infants soon stop pulling the blanket when the toy is gone; in contrast, 8-month-old infants keep pulling the blanket^5^. In the latter case, the mere presence of the blanket is sufficient to evoke the pulling behaviour that has been reinforced previously. If the action of infants is based on explicit expectations, their behaviour should instead be controlled according to behavioural goals. Their behaviour would be exhibited/inhibited depending on the expected the value of the outcome^7^,^23^. In the near future, we intend to test with our image-scratch task whether infants' spontaneous behaviour is accompanied by explicit expectations. Verification would be interesting because it strongly relates to the big issue of developmental science: how self-consciousness and self-agency emerge through the developmental process.

Several studies have shown that infants older than 1.5 years can control their actions depending on goal evaluation^7^,^8^,^23^; these infants increase/decrease the pulling action depending on the attractiveness of the reward toy. However, such a task cannot be applied to younger infants because of their immature motor ability. Our task may be a useful tool for examining goal evaluation in young infants. Although in the present study we simply demonstrate that the difference in outcome attractiveness leads to different behaviour, we can in the future conduct goal evaluation experiments with infants younger than 1.5 years using our paradigm.

Another interesting topic is that the explicit visual presentation of the gaze point as a red circle enhances spontaneous gaze control in both adults and infants. It will be important to reveal the reason underlying this finding, considering that both conditions are fundamentally identical with regard to the presence of an action–outcome association. We consider that the visual feedback of the gaze point may not only be a controllable object, but may also be an aid to attributing the effect to the self-generated action (i.e. an attribution of agency). Future studies should aim to clarify the relationship between the visual feedback of eye movement, the detection of an action–outcome contingency, and the sense of agency in both adults and infants.

We believe that the image-scratch task has great potential in developmental science because it permits quantification of infants' spontaneous behaviour accompanied by motivation regardless of differences in motor ability. We believe that our task design can be refined further and can become a powerful tool for quantifying various infants' spontaneous behaviours, including those that are organised by higher-order cognitive processes. Furthermore, our results seem consistent with results and concepts developed within constructive disciplines such as machine learning and cognitive developmental robotics^24^. Several studies have highlighted the importance and functional significance of intrinsically motivated behaviour^25^,^26^, such as behaviour driven by acquisition of novel information^27^ and goal-directed behaviour driven toward task fulfilment despite initial failure^28^,^29^. Integrating such findings into a cross-disciplinary conceptualisation seems to be a promising goal for future work, and could lead to improved hypothesis formulations and refined experimental designs. For such progress, we must evaluate the image-scratch task from an inter-disciplinary viewpoint, in light of behavioural psychology, neuroscience, philosophy, and constructive robotics.

## Methods

### Participants

Forty adults and 22 infants participated in Experiment 1: 21 adults were included in the with-gaze-point condition (mean age ± SD, 21.7 ± 3.7 years; 12 women and 9 men) and 19 adults were included in the no-gaze-point condition (mean age, 20.7 ± 1.2 years; 13 women and 6 men); 12 infants participated in the with-gaze-point condition (mean age, 8.6 ± 0.4 months; 5 females and 7 males) and 10 participated in the no-gaze-point condition (mean age, 8.9 ± 0.5 months; 4 females and 6 males).

All participants were recruited from the participant pool of Tamagawa University (Tokyo, Japan) via telephone calls or email. All adults and parents of infants gave written informed consent before participating in the study. This study was carried out in accordance with the guidelines approved by the Ethics Committee of Tamagawa University.

### Apparatus and stimuli

Eye gaze was measured using a Tobii near-infrared eye tracker (T120; Tobii Technology AB) which was integrated with a 17-inch LCD monitor. The display resolution was 1024 × 768 pixels and the sampling rate was 60 Hz. A standard 9-point calibration was used. The task programming was completed in Visual Basic 6.0 and Tobii Eye Tracker SDK.

In the image-scratch task, five pictures were presented sequentially on the display for 30 s each. Just after the presentation of a picture, the whole display was covered with a black layer. When an eye gaze was detected as a point on the display, a circular area with a radius of 50 pixels was scratched off, and the corresponding area of the picture became visible (Fig. 1A). There was no requirement of gaze fixation for the scratching.

Figure 1C shows an actual visual stimulus used in Experiment 1. To compare eye movements between the first and fourth trials, the same pictures of solid simple geometric patterns were presented in the first (Phase I) and fourth (Phase II) trials. To increase the participants' rate of exploration, colourful pictures were presented in the second and third trials. Finally, in the last trial, another colourful picture was presented, and a 10-s interruption phase was inserted into 20 s of normal operation, the interruption onset chosen randomly for each participant between 7 s and 10 s after the beginning of the trial. In this phase, the eye movements of participants did not scratch off the black layer, even if a fixation was detected (Fig. 1C). Instead, the image remained unchanged. Before and after the interruption phase, gazes scratched off the black layer as in all other trials.

To get the participants' attention, a movie with a voice saying ‘Look! Look!' was presented for 3 s before the presentation of each picture. Pleasant background music was played while the eye tracker collected the eye-movement data. The music faded out when the participant was not looking at the display. Both adults and infants executed the same task.

### Procedure

In all experiments, participants were seated approximately 60 cm away from the display and eye tracker. The adults were only instructed to look at the display, with no further instructions regarding the rules of the task. The infants, who were fastened in a baby carrier to prevent them from standing up, sat on their mothers' lap, facing the display, and completed the same task. Stimuli were presented immediately after the 9-point calibration was executed. After task execution, adult subjects were asked to complete a questionnaire asking whether they had noticed the gaze–scratch contingency and scratched off the black layer intentionally.

### Data rejection

We rejected data from those participants who gazed outside the display for more than 80% of the mean time from the first to the fourth picture and during the interruption phase. In Experiment 1, 5 adults and 16 infants were excluded from further analyses. In addition, 13 infants were excluded because they did not complete the task.

## Author Contributions

M.M. and H.T. developed the study concept and design. Testing, data collection, and analysis were performed by M.M. and H.T. M.M., H.T., M.R., H.O. and T.O. wrote the paper and approved the final version of the paper for submission.

## Supplementary Material

Supplementary Information Supplementary information

Supplementary Information Supplemental Video S1.

Supplementary Information Supplemental Video S2.

Supplementary Information Supplemental Video S3.

Supplementary Information Supplemental Video S4.

## Figures and Tables

**Table 1:** The numbers of adult and infant participants assigned to spontaneous and passive groups

	Condition	Spontaneous scratching	Passive viewing
Adults	with-gaze-point (n = 19)	16	3
	no-gaze-point (n = 17)	8	9
Infants	with-gaze-point (n = 12)	8	4
	no-gaze-point (n = 10)	1	9
