# Supplementary material for: The image-scratch paradigm: a new paradigm for evaluating infants' motivated gaze control
Source: Sci Rep. 2014 Jun 30;4:5498. doi: 10.1038/srep05498 (PMC4074783; doi:10.1038/srep05498)
Supplement: Supplementary Information [file srep05498-s1.pdf]

Supplementary information

Title: The image-scratch paradigm: a new paradigm for evaluating infants' motivated gaze control

Author list: Michiko Miyazaki, Hideyuki Takahashi, Matthias Rolf, Hiroyuki Okada, and Takashi Omori

Supplemental movies

These videos illustrate the eye trajectories of each type of participant. To show the gaze-point clearly, we substituted a white background for the colourful pictures used in the study.

**Supplemental Video S1.**	**Eye trajectories of an adult showing spontaneous scratching**
**Supplemental Video S2.**	**Eye trajectories of an adult showing passive viewing**
**Supplemental Video S3.**	**Eye trajectories of an infant estimated as showing spontaneous scratching**
**Supplemental Video S4.**	**Eye trajectories of an infant estimated as showing passive viewing**

Figure S1.

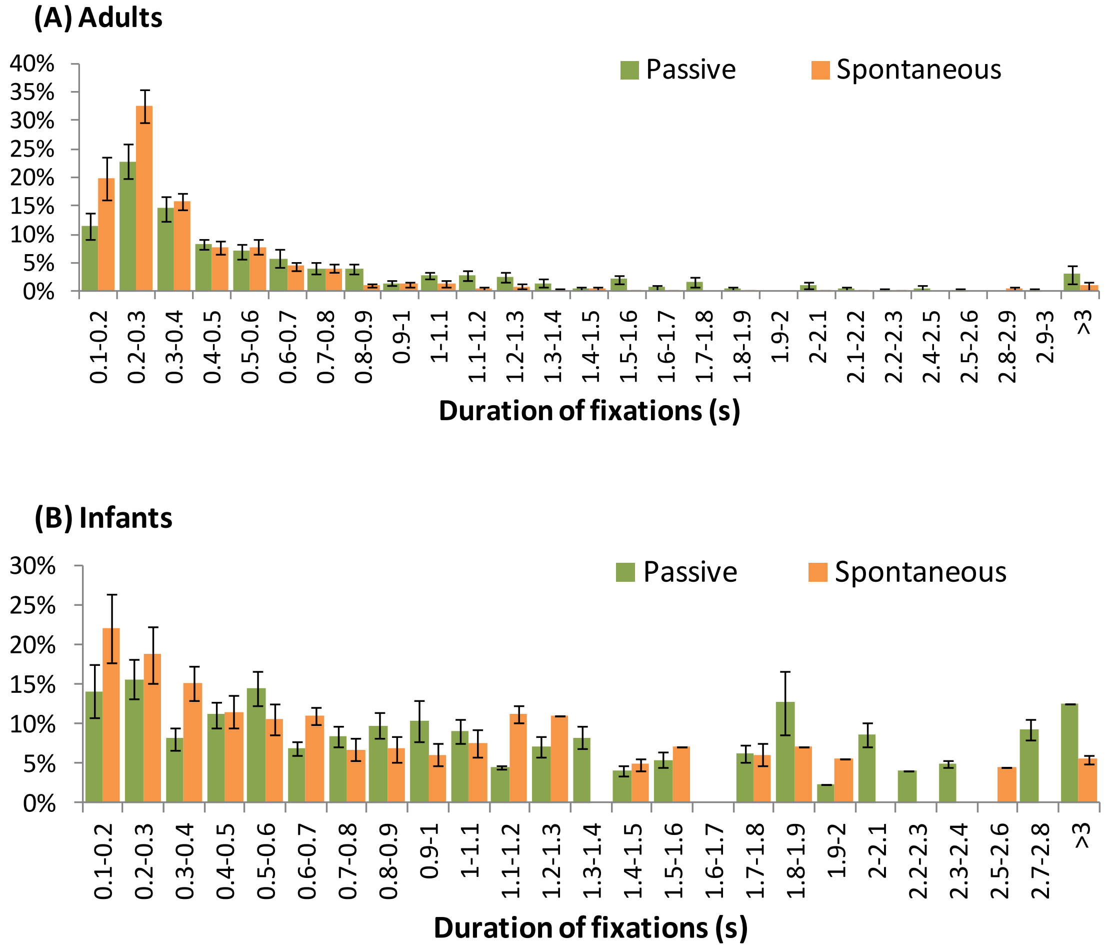

Figure S1. Duration of fixations in Phase II in (A) adults and (B) infants. The error bars indicate the standard errors. The percentage of short (less than 0.5 s) fixations in the spontaneous group is higher than that in the passive group in both adults and infants. These tendencies indicate an increase in the frequency of quick gaze shifts in spontaneous scratching, because these individuals scratch the black layer efficiently by not gazing at the same location for a long time and by quickly shifting their gaze.

Experiment 2: Control of eye movement based on outcome attractiveness

The results of Experiment 1 demonstrate that both the adults and the infants had a high sensitivity to the gaze–scratch contingency in spontaneous scratching, because they exhibited a high exploration rate during the interruption phase. The exploration rate is the best indicator of spontaneous eye movement because of its high prediction accuracy with respect to questionnaire reports in adults. This suggests that the infants who show a high exploration rate control their eye movements spontaneously.

Importantly, however, we should note that eye movements are not only influenced by motivated (intention-driven) behaviour, but also by task-driven behaviour [1], i.e., they come under the influence of various bottom-up factors, such as sensory-evoked reflex-based responses [2]. The evaluation of motivated behaviours requires their differentiation from behaviours that are influenced by automatic, bottom-up factors.

Thus, in Experiment 2, we asked whether the infants were truly motivated to scratch off the black layer and to expose the hidden pictures. To examine this, Experiment 2 used five monochromatic (grayscale) images as hidden pictures instead of colourful pictures; the task was executed by 12 new infants (mean age, 8.3 ± 0.49 months; 6 females and 6 males) in the with-gaze-point situation (non-attractive condition). In Experiment 2, data from an additional nine infants were excluded from analysis because the data of seven infants did not meet the acceptance criterion and two infants did not complete the task.

The exploration rate under the non-attractive condition was as high (non-attractive: 63.1%) as observed in the spontaneous scratching group (spontaneous: 45.1%; t [19] = 1.52; p = 0.15, two-tailed, n.s.). This result suggests that infants had a high sensitivity to the gaze–scratch contingency. Interestingly, however, the size of the scratched area did not differ between Phases I and II in the non-attractive condition (Phase I, 22.7%; Phase II, 24.6%; t [11] = −0.64; p = 0.54, two-tailed, n.s.). These results suggest that infants were not motivated to expose the hidden pictures under this condition and did not show gaze control of eye movement because grayscale images were not as attractive as colourful images.

Experiment 3: Influence of gaze-scratch contingency (yoked control)

The results of Experiments 1 and 2 suggest that the infants who were categorised as spontaneous showed motivated control of eye movement. However, additional factors should be investigated before concluding that the spontaneous infants are able to show motivated control during the image-scratch task. The saliency of visual images could possibly have influenced their gaze pattern. For example, most infants categorised as spontaneous were continuously exposed to a red circle indicating their gaze point during the image-scratch task. The red circle could possibly have evoked reflex-based gaze and led to the different gaze pattern of these infants. In Experiment 3, we aimed at examining the effect of saliency of the visual images on the image-scratch task.

To examine whether infants were truly influenced by the gaze-scratch contingency instead of mere visual saliency, we performed a yoked control experiment using another group of infant participants. Nine infants participated in the non-contingent condition (mean age, 8.2 ± 0.4 months; 7 females and 2 males). An additional 20 infants were excluded from analysis because the data of nine infants did not meet the acceptance criterion and 11 infants did not complete the task. The non-contingent condition consisted of the presentation of a movie that showed exactly what had been on the screen while other infants who had successfully detected the action–outcome contingency (infants in the spontaneous scratching group) were performing the task. Although the infants in the non-contingent condition were not able to detect the gaze–scratch contingency, they watched stimuli that were completely consistent with those that had been watched by the spontaneous infants during the task.

If visual saliency of the red circle that indicated the gaze point raised the exploration rate in the spontaneous infants, the exploration rate in the non-contingent condition should also be higher. However, the exploration rate in the non-contingent condition was significantly lower than in the spontaneous infants (non-contingent, 18.8%; spontaneous, 45.1%; $t\,[8] = 6.68$, $p = 0.00016$, $r = 0.92$, two-tailed). This result suggests that the eye movements of the spontaneous infants did not depend on the saliency of visual features such as the red circle.

1 Rowlands, M. *Body Language: Representing In Action*. (MIT Press, 2006).
2 Michel, F. & Anderson, M. Using the antisaccade task to investigate the relationship between the development of inhibition and the development of intelligence. *Dev. Sci.* **12,** 272-288 (2008).
